# Medial Temporal Tau Pathology Is Associated With Verbal Memory

**DOI:** 10.1093/geroni/igaa057.2770

**Published:** 2020-12-16

**Authors:** Murat Bilgel, Jacob Ziontz, Andrea Shafer, Luigi Ferrucci, Dean Wong, Susan Resnick

**Affiliations:** 1 National Institute on Aging, Bethesda, Maryland, United States; 2 Johns Hopkins University School of Medicine, Baltimore, Maryland, United States

## Abstract

Medial temporal tau pathology is frequently observed in individuals over 70 regardless of cognitive status. To understand the link between tau and cognitive performance, we evaluated tau pathology using 18F-flortaucipir positron emission tomography among 95 cognitively normal participants from the Baltimore Longitudinal Study of Aging. We examined tau levels in early Braak regions (entorhinal cortex and hippocampus) in relation to verbal episodic memory performance concurrent with and prior to the tau scan using linear mixed effects models adjusted for age, sex, amyloid status, and time from PET scan. Higher hippocampal tau burden had a trend-level association with lower concurrent memory performance (p=0.05). Greater tau pathology in the hippocampus and entorhinal cortex was associated with steeper decline in memory performance prior to tau scan (p=0.013 and 0.026, respectively). These findings suggest that therapeutic interventions targeting tau pathology may need to be administered early among cognitively normal individuals to prevent memory decline.

